# A novel proxy for energy flux in multi-era wildfire reconstruction

**DOI:** 10.1038/s41598-024-78219-3

**Published:** 2024-11-02

**Authors:** Thomas Theurer, Dmitri Mauquoy, Rory Hadden, David Muirhead, Zakary Campbell-Lochrie, Sergio Vargas Córdoba, Clemens von Scheffer, Daniel Thomas Coathup

**Affiliations:** 1 School of Geosciences, University of Aberdeen, Aberdeen, UK; 2 School of Engineering, University of Edinburgh, Edinburgh, UK

**Keywords:** Wildfire, Climate change, Charcoal, Raman spectroscopy, Energy flux, Peatland, Carbon cycle, Climate change, Palaeoclimate, Energy, Organic chemistry, Physical chemistry, Biogeochemistry, Climate sciences, Ecology, Natural hazards, Engineering, Raman spectroscopy

## Abstract

Escalations in wildfire activity are of significant global concern, particularly within vulnerable wetland ecosystems integral to natural carbon sequestration and climate change mitigation. Our understanding and management of future wildfire activity may be better contextualised through the study of historic and ancient fire records, independent of human influence. Methods of study include ‘geothermometry’ - approximating ancient fire intensity from temperature-dependent changes in the chemistry of fossil charcoal. Though well established in their relation to experimental charcoalification, these methods still fail to quantify the true intensity of ancient fires, as a measure of energy release. As a result, their applicability, and contributions to the characterisation of modern fire activity, remain uncertain. Here, we present a novel measure of wildfire energy release, as a proxy for true intensity, through the co-application of cone calorimetry and Raman spectroscopy of charcoal. By applying a range of wildfire heat fluxes to variable peatland fuel mixes, this research demonstrates the complexity in correlating fire behaviour and charcoal microstructure. Further statistical analyses suggest a correlation between spectroscopic results, measures of CO and CO_2_ release, and fire severity. This offers a principal measure of ancient wildfire intensity, consistent with modern practices in wildfire modelling, monitoring, and management.

**Supplementary Information:**

The online version contains supplementary material available at 10.1038/s41598-024-78219-3.

## Introduction

Wildfire disturbances may be considered one of the most powerful demonstrations of our rapidly changing global climate, as fire-promotive conditions (‘fire weather’) develop further^1–3^. Boreal regions are suffering substantial increases in wildfire activity^4^, now under a fire regime otherwise unprecedented in the past 10,000 years^5^. Observations of over-wintering behaviour and re-intensification in smouldering fires, known colloquially as ‘zombie fires’, raise further concerns as to the stability of peatlands and permafrost under accelerated high-latitude warming^6–8^. Peatlands, prevalent in the boreal zone, represent a major terrestrial carbon store and natural sink for atmospheric CO_2_, reflecting a greater reserve of carbon than the global cumulative mass of live vegetation^9^. Their persistence and capability for storage are limited by an increasing susceptibility to direct (e.g., agricultural practices, draining) and indirect (e.g., rising atmospheric temperatures, shifting rainfall patterns, increases in shrub canopy) anthropogenic disturbances, including wildfire, e.g.,^10–15^. There has never been a more pressing need to understanding the role of climate change in modifying fire activity and earth-system feedback.

Contemporary efforts in climate study, prediction, and mitigation planning have turned to pre- and deep historic records to understand future scenarios in climate change^16^. Trends in palaeowildfire behaviour during ancient periods of climate hyperthermal - referring here to periods of rapid, extreme climatic warming (e.g., Palaeocene-Eocene Thermal Maximum) - and their relation to modern climate and ecosystem flux, have also been investigated^17^. In the study of multi-era (i.e., decadal, centennial, and millennial timescales) changes to fire and climate interactions, peatland records are particularly beneficial - attributed to their continual, highly resolved records of biomass and charcoal^18–20^ which can be readily radiocarbon dated^21^.

Thermometry is a contemporary concept in palaeowildfire study whereby formation temperatures are determined from palaeocharcoals, as a proxy for ‘wildfire temperature’ and intensity^22^. To achieve this, a variety of geochemical methods have been applied to the progressive (experimental) pyrolysis of charcoal, often derived from small-scale, furnace applications, e.g.,^23^. Temperatures experienced by a fuel during combustion instead represent a complex array of co-interactions between vegetation characteristics and the incidence, absorption, and release of thermal energy^24^. This complexity, and the nature of energy flux in wildfire as a true measure of fire intensity^25^, has yet to be fully investigated, nor utilised in studies of charcoal geothermometry. Recent studies in Raman spectroscopy^26^, Fourier-transform infra-red (‘FT-IR’) spectroscopy^27,28^ and charcoal reflectance^29–32^ continue to reiterate this. Quantitative reconstruction of palaeowildfire intensity from palaeocharcoals as a function of energy release, however, remains severely underexplored.

Raman spectroscopy - as a rapid, versatile, non-destructive technique - is well placed to explore this potential. In applying Raman spectroscopy, charcoal is characterised by its microscopic structure (‘microstructure’ *herein*), comprising carbon-carbon bonding within polyaromatic crystallite units^33–35^. First order Raman bands ‘D’ and ‘G’ are indicative of carbon-carbon vibrational modes A_1G_ and E_2G_, respectively^33,34^. These bands and their derivative parameters indicate a microstructural heterogeneity within charcoals that reduces with charcoalification under increasing temperature^36,37^. We expect that this principle is consistent with increasing heat (energy) flux incident on the burning material. However, differences in fuel composition and response, and the volatility of flaming combustion, suggest a limitation to the translation of fire behaviour to Raman spectra.

Here, we investigate the response of common Raman parameters to charcoals, produced from different intact peatland surface fuel mixes (‘mesocosm’ *herein*) under variable wildfire-replicative heat fluxes. By defining thermal exposure as the incident heat flux (kW/m^2^) applied until natural flameout (with 30s simulated residual burning), we have considered combustion behaviour solely as a function of vegetation characteristics. This reflects the closest, practical (experimental) approximation of field-scale conditions, considerate of the aforementioned complexities in energy flux-fuel interactions. Results show a strong dependence of Raman band separation, intensity- and area ratios on fuel composition and heat flux. In the reconstruction of heat flux from experimental charcoals, other parameters show diagnostic limitations, resulting from deviations in trend, a feature that we attribute to the loss of charcoal mass during combustion. By applying complementary statistical analyses to the assessment of (i) differences between Raman parameters over increasing incident heat fluxes (ANOVA, Bayesian ANOVA); (ii) multi-dimensional co-interactions between calorimetric and spectroscopic data (Permutational ANOVA, Cluster Analysis, Principal Component Analysis); and (iii) correlative significance (Spearman’s Rank, Generalised Least Squares Regression), we have evaluated the capability of Raman spectroscopy in reconstructing features of combustion from charcoalified plant material. From these data, we offer estimations of total incident energy, energy release, and wildfire intensity over multiple timescales. This is the first method developed that quantifies energy-dependent palaeowildfire intensity, consistent and compatible with modern principles in wildfire characterisation.

## Results

The multi-scale variability of peatland vegetation (changes in the mix of mosses, dwarf shrubs and graminoids through time) suggests a limitation to any consistent and quantifiable relationship between fire heat flux and microstructural change in charcoals. This limitation may therefore extend to a reliable proxy method of wildfire calorimetry. This is supported by the observation that combustion behaviour^38,39^, physiochemical change^36,40^, preservation potential^41,42^ and spectral response (e.g., Raman spectroscopy^43,44^ and FT-IR^28^) are strongly dependent upon the chemistry and structure of precursor materials during charcoalification. It is important, therefore, to consider the response of variable peatland compositions under different heat flux treatments (20–80 kW/m^2^). Therefore, mesocosm compositions under calorimetric experimentation, *as per* this study, include samples comprising of > 75% mosses (MS_> 75_), > 75% graminoids (GR_> 75_), > 75% dwarf shrubs (DS_> 75_), and 25–50% mixes dominated by mosses and graminoids (MSGR_Mix_), dwarf shrubs and mosses (DSMS_Mix_), and dwarf shrubs and graminoids (DSGR_Mix_).

**Figure 1:**
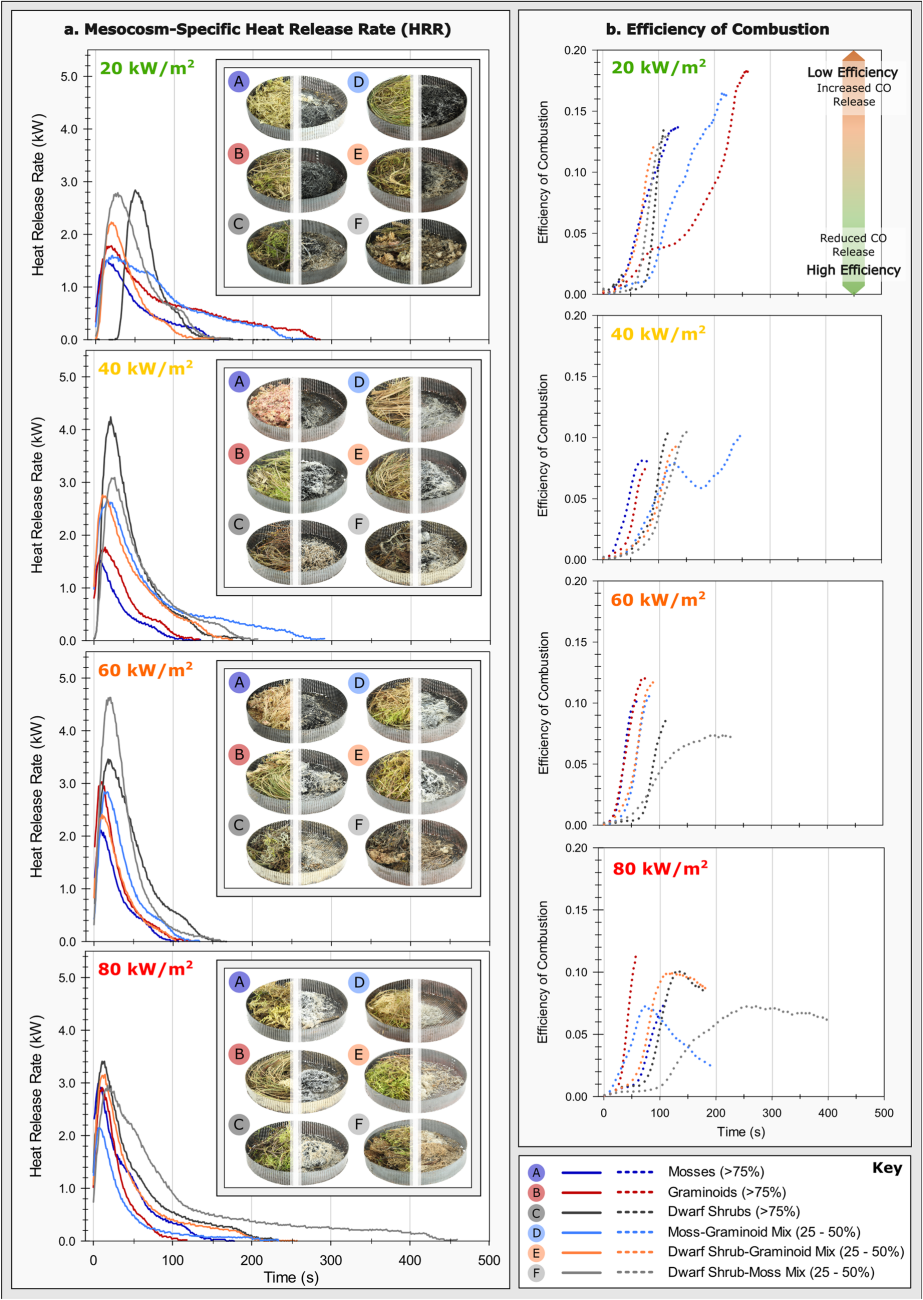
Data plots detailing (**a**) trends in heat release rate (kW), and (**b**) efficiency of combustion (as a proportion of CO to CO_2_ yield) for the mesocosm compositions, across four fire-replicative heat fluxes (20, 40, 60, and 80 kW/m^2^), respectively. Images of mesocosm samples prior- (left) and subsequent to (right) calorimetric treatment are shown adjacent to the respective heat release rate plot (**a**).

**Figure 2:**
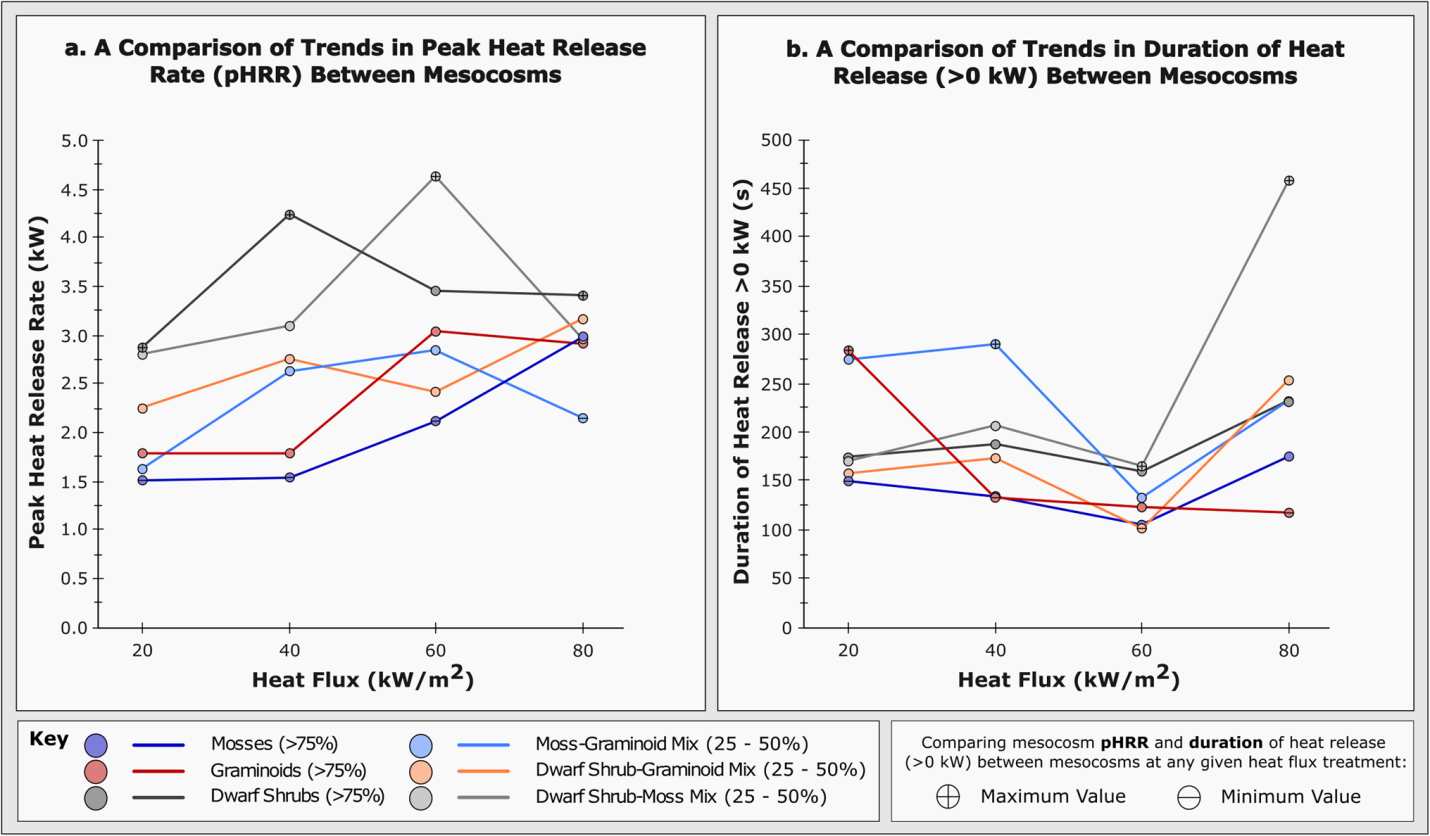
Data plots reiterating comparative trends in (**a**) peak heat release rate (kW), and (**b**) duration of heat release > 0 kW, between mesocosm compositions, across four fire-replicative heat fluxes (20, 40, 60, and 80 kW/m^2^) as per Fig. 1a. ‘Minimum’ and ‘maximum’ values at each heat flux application are shown accordingly.

**Figure 3:**
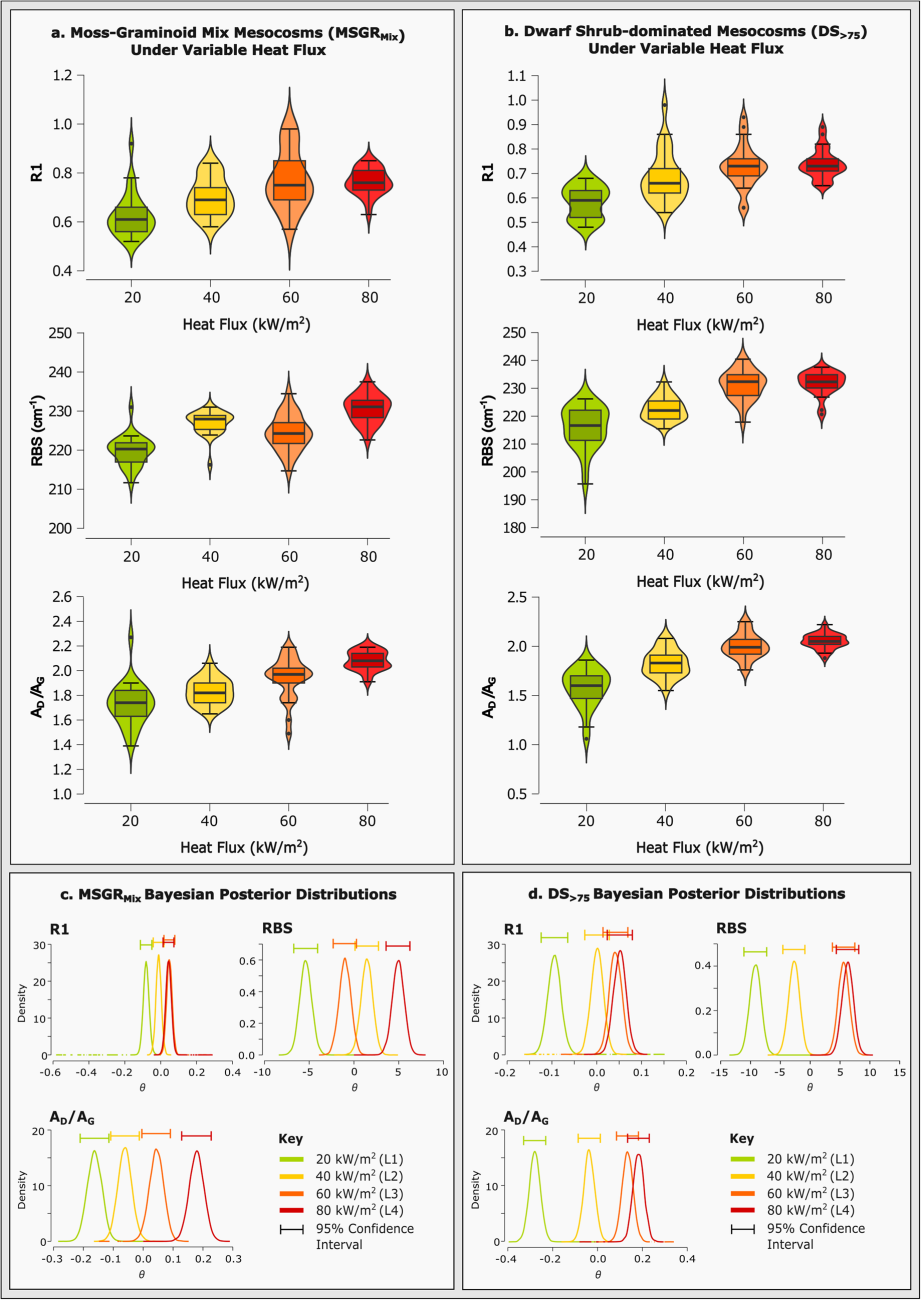
Data plots **a**, **b**) demonstrating the behaviour of Raman spectral parameters (R1, RBS and A_D_/A_G_) for moss-graminoid and dwarf shrub mesocosms over four wildfire-replicative heat fluxes (20, 40, 60, and 80 kW/m^2^), and **c**, **d**), respective Bayesian posterior distributions indicating credible intervals at experimental heat fluxes, relative to whole-data mean values (ϴ).

It is also beneficial to consider these data in relation to the behaviour of fuels during combustion, expressed here as measures of heat release rate (kW), burning duration (s), and peak heat release rate (kW). To this effect, we report a corresponding variability in fire behaviour (Figs. 1 and 2) and spectral response (Fig. 3 and Supplementary Table S3). Reference, herein, to ‘minimum’ or ‘maximum’ peak calorimetric values corresponds to the comparison between mesocosm data at any given incident heat flux (Figs. 1 and 2).

### Combustion behaviour in peatland mesocosms

Considering time-resolved heat release rate (Fig. 1), inconsistent patterns of combustion emerge with increasing incident heat flux, both within and between mesocosm compositions. A systematic increase in minimum peak heat release rate (‘pHRR’ *herein*) independent of mesocosm classification occurs from 20 to 80 kW/m^2^ (Fig. 2). This accompanies a peak, and subsequent inversion, in maximum pHRR and range of pHRR values at 40 kW/m^2^ across the tested mesocosms. Reduced variability in pHRR range between mesocosms at 80 kW/m^2^ indicates a threshold at which constituent composition no longer becomes the primary factor in determining heat release behaviour. Minimum durations of heat release (*as per* a threshold > 0 kW) remain broadly consistent across tested heat fluxes (~ 100–200 s), whilst the variability in duration between mesocosms at each heat flux increases up to 80 kW/m^2^ (Fig. 2). This is limited by a substantial consistency in heat release response between mesocosms at 60 kW/m^2^. Maximum pHRR values across the applied heat fluxes are consistently associated with dwarf shrub (DS) material, including some limited moss (MS) inclusion (DS_> 75_ and DSMS_Mix_). Mesocosms that generate minimum pHRR values and prolonged heat release, as observed in Figs. 1 and 2, often contain moss and graminoid (GR) material (MS_> 75_, GR_> 75_, MSGR_Mix_, and DSMS_Mix_). Increased pHRR is therefore a feature of combustion commonplace to the inclusion of dwarf shrub material, relative to other mesocosm compositions under equivalent incident heat fluxes.

By determining the efficiency of combustion with time (Fig. 1b), as a function of the proportion of CO yield (g/g) to CO_2_ yield (g/g) during calorimetry, assumptions may arise as to the combustion behaviour of fuels. Generally, the mesocosms tested under increasing incident heat flux indicate a trend of rapidly reducing combustion efficiency as the duration of carbon release increases. Efficiency of combustion shows an overall increase from 20 to 40 kW/m^2^, followed by a reduction at 60 kW/m^2^, and an increase again up to 80 kW/m^2^. At 20 kW/m^2^, the decrease in efficiency is more gradual for GR_> 75_ and MSGR_Mix_, whereas efficiencies at 40 kW/m^2^ show a remarkable consistency in trend, if not for the limited inversion in efficiency at ~ 120s for MSGR_Mix_. Similarly, at 60 kWm^2^, trends in carbon release differ only for DSMS_Mix_, displaying a gradual reduction in efficiency that plateaus after ~ 180s. When treated under 80 kW/m^2^ heat flux, mesocosm samples indicate increased variability in CO/CO_2_ release, as a proxy for efficiency. Though MS_> 75_ and GR_> 75_ indicate a rapidity in the reduction of efficiency with time, as with other heat flux treatments, DS_> 75_, MSGR_Mix_ and DSGR_Mix_ all show a marked increase in efficiency until extinguishment.

### Raman response under variable heat flux

Similar fuel-dependent variabilities are found within the Raman parameter dataset. Progressive charcoalification is typically associated with a reduction in parameters dependent upon full-width at half-maximum (‘FWHM’) values from the D- and G-band (D-FWHM, G-FWHM), including the width ratio ‘FWHMRa’. These spectral changes are accompanied, in turn, by an increase in D-/G-band intensity (R1) and area (A_D_/A_G_) ratios, and a growing band separation (RBS) as D- and G-bands shift dichotomously^45–47^. These trends, evident in *Supplementary Figure **S1*, are generally replicated with increasing heat flux across the tested mesocosms, offering moderate-high linearity in trend, particularly for applications of R1, RBS, and D-band position. This is contrasted by a predominance of poor trend linearity for MS_> 75_ and GR_> 75_mesocosms, irrespective of the applied parameter. Similarly, FWHMRa trends are inverse to those expected across the mesocosms^26^: a result of non-linearity in constituent D- and G-FWHM trends. This is a feature most prominent in mesocosms dominated by graminoid, and often moss, material (MS_> 75_, GR_> 75_, MSGR_Mix_, DSGR_Mix_, and DSMS_Mix_*as per* Supplementary Figure S1). The applicability of Raman parameters in determining heat flux from charcoalified plant material shows a clear dependence upon the fuel type.

There is a definitive influence of composition – namely mosses and graminoids – upon lower pHRR, prolonged heat release, increased data variability, and trend inversion for Raman parameters (Figs. 1, 2 and 3 and Supplementary Table S3).

### Quantifying fire heat flux from Raman spectra

Of the parameter-mesocosm trends considered above, R1, RBS and A_D_/A_G_ each offer a positive, linear relationship with incremental heat flux when applied to mesocosms DS_> 75_ and MSGR_Mix_ (Fig. 3). D-band position, similarly, offers a strong linear (inverse) trend with heat flux for DS_> 75_ and MSGR_Mix_. It is, however, limited in its applicability and robustness as a parameter, on account of one-dimensionality - discounted from this study as a result. All parameters record a bimodal response in the data at each respective heat flux, as R1 and A_D_/A_G_ are typically skewed to lower values, whereas RBS is skewed to higher values, relative to the median (Fig. 3).

The calorimetric capabilities of R1, RBS, and A_D_/A_G_, as applied to DS_> 75_ and MSGR_Mix_, are further supported by analyses of variance (ANOVA) as per *Supplementary Table S4*, suggesting statistical significance between all mean values at respective heat fluxes. This is coupled with high eta-squared effect sizes across the mesocosm-parameter combinations. Within these combinations, pairwise post-hoc comparison (Tukey and Games-Howell) suggest non-significance is predominant between 40 and 60 kW/m^2^ and 60–80 kW/m^2^ median data. Non-significant Levene’s test values for R1 applied to DS_> 75_, and RBS and A_D_/A_G_ applied to MSGR_Mix_, suggest dataset non-normality of variance is not specific to the mesocosm or parameter in application (Supplementary Table S4).

Further Bayesian ANOVA statistical modelling suggests that the application of R1, RBS, and A_D_/A_G_ to DS_> 75_ and MSGR_Mix_ yields high (extreme—strong evidence) Bayes factor effect sizes, universally (Supplementary Table S5). This is supported by low model error (< 0.002%). Bayes factors further indicate that A_D_/A_G_ and RBS are best placed to accurately model heat flux in dwarf-shrub dominated and moss-graminoid mix mesocosms, respectively. However, mesocosm-specific models of heat flux are limited by credible interval overlap, often occurring between 40 and 60 kW/m^2^ and 60–80 kW/m^2^, *as per* model averaged posterior summary (Supplementary Table S6).

The application of mesocosm-specific heat flux derivation, using Raman, may be further limited by issues of time-consuming preparation, unconscious sampling bias, uncertainty in (fuel) identification^48^, and the incompatibility between ordinal datasets and robust regression modelling. Multi-era fire activity in peatlands may therefore be characterised more effectively through ‘universal calorimetry’ – considering calorimetric data non-specific to precursor fuels. We also recommend considering heat flux as a function of incident heat flux, integrated over the total duration of heat release (‘total incident energy’ *herein*) to account for variabilities in combustive behaviour, and in support of regression modelling.

### Establishing universal Raman palaeo-calorimetry and further derivatives

**Figure 4:**
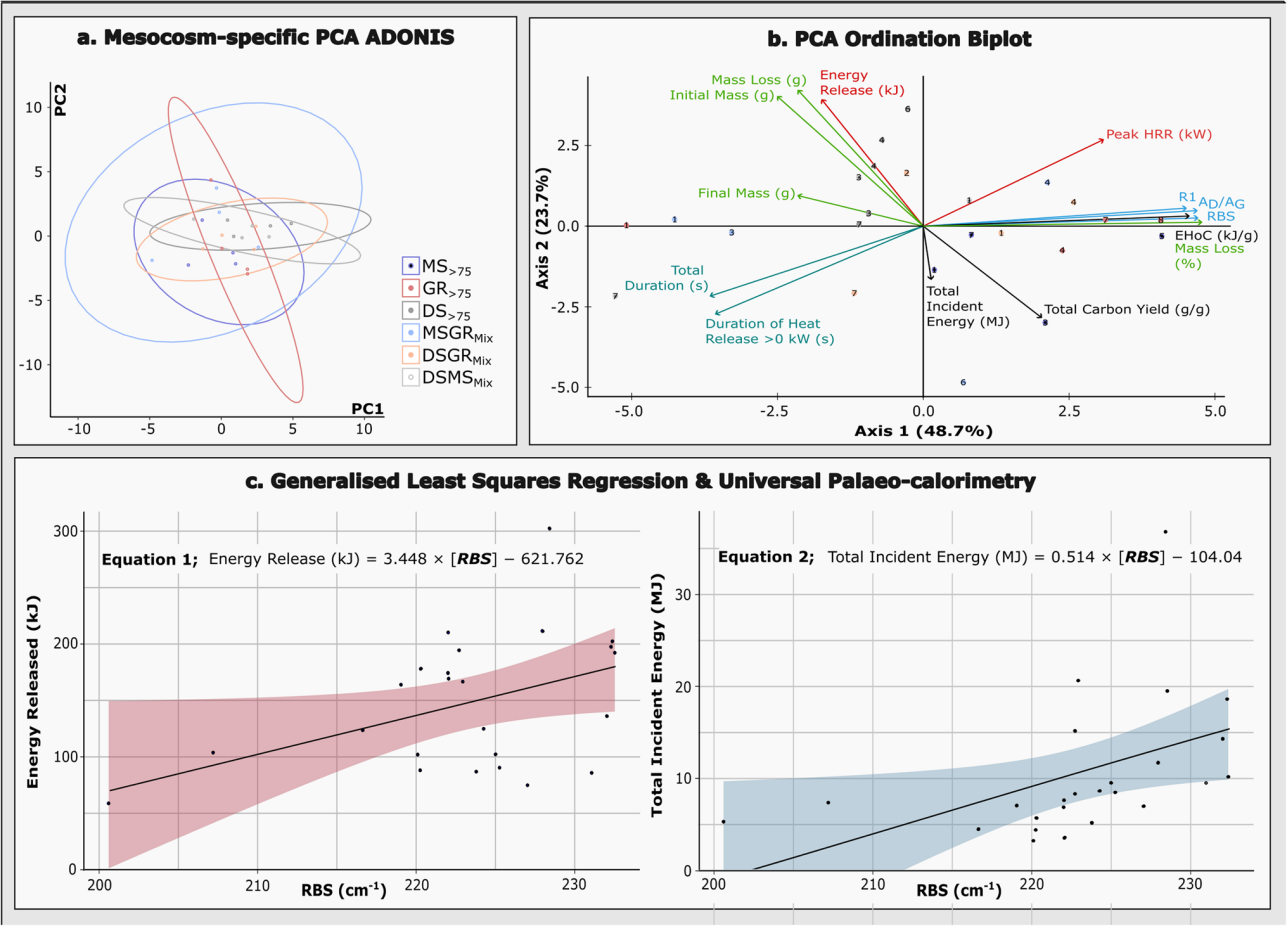
Statistical analyses, including (**a**) PCA ADONIS (*sans* Raman data) as an indication of the non-significant permutational multivariate analysis of variance between mesocosm-specific calorimetry datasets (*F*(5, 18) = 1.861), (**b**) a PCA ordination biplot demonstrating multi-dimensional relationship modelling of calorimetric and Raman (RBS, R1, and A_D_/A_G_) data (Hellinger transformed), relative to heat flux-treated mesocosm samples, *as per* panel (**a**). Here, ‘EHoC’ refers to effective heat of combustion. Panel (**c**) indicates generalised least squares regression modelling for energy release (kJ) and total incident energy (MJ), relative to whole-data Raman band separation (RBS) values. Credible intervals are shown by colour regions, and derivative equations are expressed adjacent to the respective regression plot.

Universal calorimetry is supported by a permutational analysis of variance (PERMANOVA) and Adonis cluster plotting of these data (Fig. 4a-b), indicating considerable overlap and a non-significant difference between the mesocosm datasets. This is contrary to assumptions of a fuel dependence in Raman spectroscopy, though it permits a consideration of the bulk dataset independent of compositional subdivision (∑).

Principal component analysis of calorimetric and Raman data, as presented in this study (Fig. 4b), suggests that 72.4% of dataset variance is accounted for by axes 1 (48.7%) and 2 (23.7%). Under a broad consideration of PCA structure, we attribute these axes to *fuel mass loss* and *duration of combustion*, respectively. As per the ordination, strong direct correlations are observed between groupings (a) mass loss (g), initial mass (g), and energy release (kJ); (b) total duration of calorimetry (s), and duration of heat release rate exceeding 0 kW (s); and (c) percentage mass loss, effective heat of combustion (kJ/g), R1, RBS, and A_D_/A_G_. In contrast, peak heat release rate (kW) and duration of burning (total- and heat release rate > 0 kW) bear no discernible relationship to initial mass (g), mass loss (g), or energy release (kJ). Negative correlations are observed between ordination groupings ‘a’ and ‘c’, and ‘b’ and ‘c’, as denoted above. This hints at a disparity between thermal maturation in charcoals (e.g., R1, RBS, and A_D_/A_G_) and the drivers of thermal maturation (e.g., increased energy release and prolonged burning) as hypothesised here.

Spearman’s rank correlations indicate further statistical significance between both RBS_∑_ and A_D_/A_G∑_, and percentage mass loss and total incident energy (MJ) collectively (Supplementary Table S7). Pairwise significance between R1_∑_, RBS_∑_, and A_D_/A_G∑_ reaffirms the robustness of Raman spectroscopy as an indicator of thermal maturity in charcoals. Additional statistical relationships are observed between percentage mass loss and total incident energy, and between total incident energy and total carbon yield (as CO + CO_2_ g/g). Of these relationships, generalised least squares regression modelling (see Supplementary Data) indicates a significant linear (positive) regression between RBS_∑_ and (1) energy released (kJ), and (2) total incident energy (MJ) (Fig. 4c) only, denoted as:

1 $$Energy\;Released\;\left(kJ\right)=3.448\times\left[RBS\right]-621.762$$

2 $$Total\;Incident\;Energy\;\left(MJ\right)=0.514\times\left[RBS\right]-104.04$$

## Discussion

Here, we have assessed the coherence of combustion behaviour through multiple calorimetric outputs (see Fig. 4b), including mass and energy fluxes, and Raman spectra from charred peatland fuel mixes, generated under variable heat flux calorimetry. As a result, we have identified a commonplace non-linearity, bimodality, and variability across Raman data and parametric trends. This emphasises the inapplicability of charcoal (temperature-derived) thermometry in accurately quantifying wildfire intensity. Supported by a statistical-invariability in calorimetric data, we have instead devised a method of universal ‘palaeo-calorimetry’. These data show that Raman band separation (RBS) values, collected from modern and palaeo- charcoals, equate to both quantitative energy release and total incident energy, as generated during any wildfire event. Indeed, the universality of this method and persistence of the charcoal record supports the application of Raman palaeo-calorimetry in fire reconstruction across not only historic and pre-historic records, but in the derivation of ancient fire regimes, also.

Calculated simultaneously as a function of RBS, the observed intercorrelation of incident and released energy suggests the relative coherence of an external radiant heat flux (*Q”*_E_*as per*^24^) and surface-imposed heat fluxes, as derived from a flame (*Q”*_F_*as per*^24^). Simply, as energy release increases, the energy release imparted from a flame to the surface of a material will also increase, as will radiative losses (*Q”*_L_*as per*^24^). This method therefore remains applicable and robust, irrespective of the source of thermal energy. It is also clear that there is a close association between total heat flux and the thermal alteration of the carbonaceous microstructure within charcoals during formation. This supports the measurement of wildfire heat fluxes as a convenient representation of material (e.g., fuel) exposure to a flame, and the applicability of Raman spectroscopy in defining this.

Total incident energy, as a measure of wildfire intensity, is notably susceptible to changes in inputs and outputs, for example, as the result of an environmental or material change. Accounting for these variables remains complex, often limited by uncertainty surrounding the ‘dose response’. However, as a metric, total incident energy proves highly valuable, in so far as it is easily measured during field experimentation. This offers greater opportunity for ground-truthing empirical observations and conclusions as to the behaviour of palaeowildfires.

Modern fire activity is typically characterised through computational modelling, derived from empirical calculations of fire behaviour^49^, computational fluid dynamics (‘CFD’)^50^ and integrated remote sensing, the origins and development of which are well encapsulated elsewhere^51,52^. These often rely on the integration of data such as fuel type, topography, fuel density, obstacles, and measurements of radiant heat flux^53^. Predictions may then be made as to the intensity (i.e., spatially-resolved energy release^25^), severity (i.e., fuel consumption as a measure of environmental impact^25^), area burned, rate of spread, and emissions from a wildfire event, contributing toward assessments of risk to the environment (e.g.,^54–56^), infrastructure (e.g.,^57–59^), and human life (e.g.,^60^), as well as post-fire impacts (e.g.,^61,62^). This association, and the wider implications for vegetation characteristics and propagative conditions, suggests the Raman-derivation of wildfire heat fluxes presents an opportunity for educated inference into palaeoenvironmental characteristics and their interaction with fire. This is further emphasised by PCA ordination of calorimetric and Raman data, presented here (Fig. 4b), indicating inherent, direct relationships (though non-significant at this time) between Raman parameters, carbon release, and percentage fuel mass loss.

**Figure 5:**
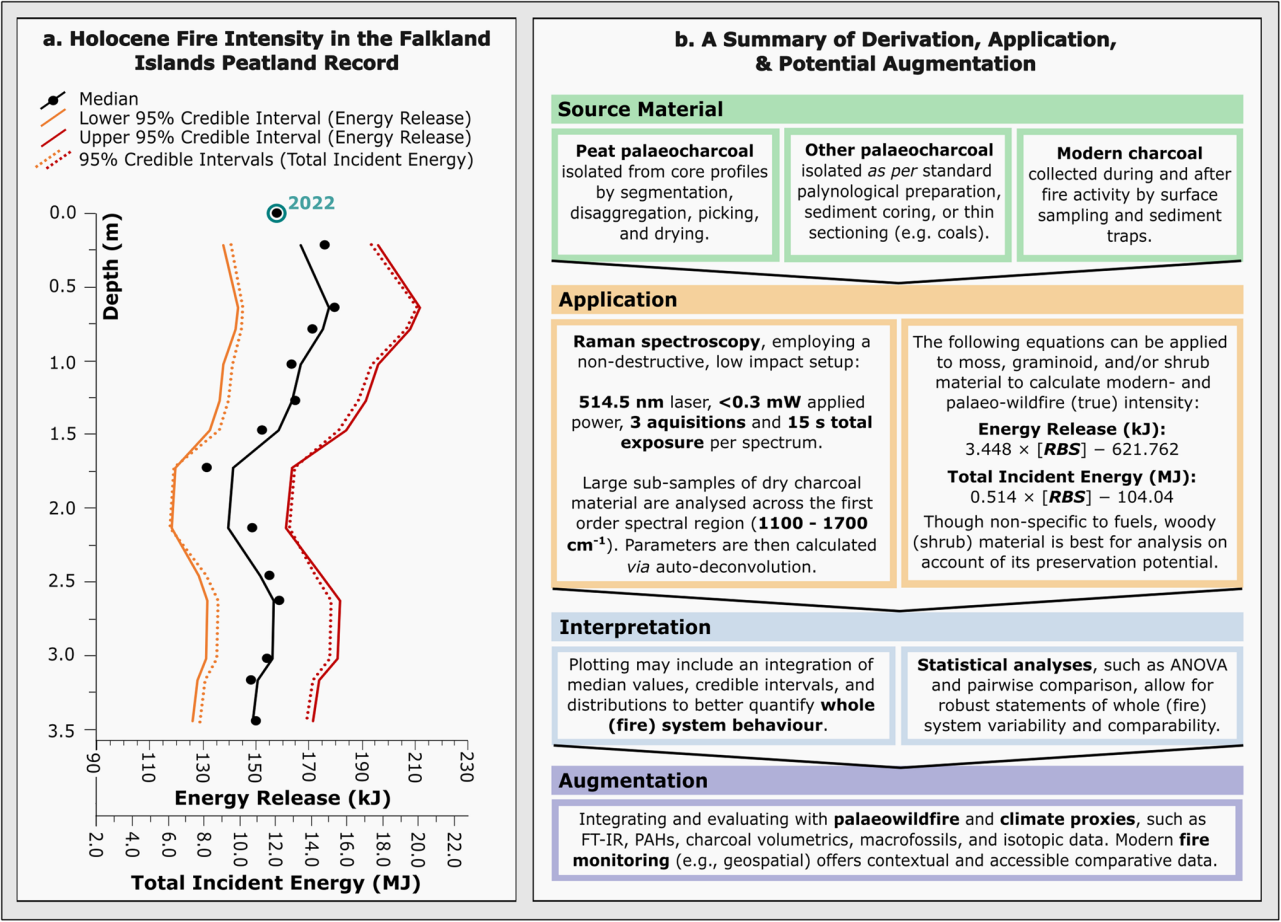
Diagrams indicating (**a**) changes in Holocene fire intensity (as per RBS-derived ‘energy release’ and ‘total incident energy’ calculations) with depth across a peat core profile from the Falkland Islands. Median and credible interval trends are shown here as two-period moving averages. Panel (**b**) offers a summary of the Raman palaeo-calorimetric method, as determined in this study, with recommendations for the application, interpretation, and augmentation of subsequent data.

The method presented here (Fig. 5b) remains the first and only quantitative measure of energy in palaeowildfire, as a proxy for true intensity that remains compatible with contemporary wildfire modelling^53^. This method, in turn, resolves limitations to historic charcoal geothermometry, namely, the validity of experimental pyrolysis and ‘fire temperatures’, and their incongruence with true fire intensity and severity.

Modern assessments of wildfire heat flux are, however, limited by issues of environmental heterogeneity and the translation of empirical understanding to macro-scale processes^63–68^. This offers an opportunity to calibrate modern methods of heat flux modelling via post-fire charcoal sampling and Raman calorimetry. With this, comes uncertainty in accurate characterisations of palaeowildfire activity, at scale. Further uncertainty lies in the apparent influence of vegetational fuel type on combustion behaviour and spectral presentation. Moss and graminoid inclusion show a definitive influence over reduced pHRR, prolonged heat release, enhanced microstructural variability, and trend inversion during experimental calorimetry. Our removal of fuel moisture content (FMC) prior to calorimetry, as a principal determinant of combustion behaviour, suggests a greater influence from fuel morphology and chemistry^69^. Further experimentation may facilitate the inference of fuel moisture and climate from modern and palaeo-calorimetric results.

The presentation of microstructural maturity in charcoals, and therefore the recorded intensity of a fire event, is seemingly dependent upon fuel composition, heat flux, and sampling procedures. For instance, under limited heat flux (e.g., 20 kW/m^2^) all fuels, including those considered less resistant to complete combustion (e.g., graminoid, moss) are more likely to be preserved under restricted mass loss^70^ due, in part, to lower bulk density^42^. As a result, charcoals are (a) more easily identifiable, compared to partially charred or uncharred material^40,71^, and (b) more likely to accurately ‘record’ a microstructure associated with peak 20 kW/m^2^ flux. Data variability between 40 and 60 kW/m^2^ is undoubtedly related to variabilities in both the preservation of material and accurate microstructural representation of the applied heat flux. These variabilities are then transferred to spectral sampling, whereby all material appears charred, though is ultimately variable in microstructural maturity. At 80 kW/m^2^ the proportion^72^ and rate of mass loss^71,73^ experienced is instead great enough to consume the majority of charcoal mass. As a result, only those fuels that have, in some capacity, remained insulated from the most intense heat are preserved. The presentation, therefore, of heat flux at 80 kW/m^2^ is likely understated, and indicative of the source of trend inversion or plateau in Raman data under variable heat flux.

In contrast, heat flux treatments 40 and 60 kW/m^2^ appear to generate the most consistent trends in combustion efficiency across the mesocosm samples tested. This trend of increasing consistency up to 60 kW/m^2^ is matched by a similar trend in the rapidity of the reduction in combustion efficiency with duration of carbon release. This is consistent with periods of increasing smouldering combustion, as a source of progressive CO release. Smouldering combustion, and the subsequent consumption of fuels, may therefore account for the variability in Raman data observed between 40 and 60 kW/m^2^. The source of disparity in combustion efficiencies between mesocosms at 80 KW/m^2^ is, however, less clear. For now, we attribute this observation to the complex interplay of fuel chemistry and mix, pyrolysis rate, ignition behaviour, and subsequent combustion behaviour.

To demonstrate the potential and accessibility of Raman palaeo-calorimetry, we have applied Eqs. (1) and (2) (as above) to palaeocharcoals isolated from a peat core profile, collected from the Falkland Islands (Supplementary Table S8). As highlighted in Fig. 5a, energy release and total incident energy values are indicative of dramatic wildfire intensification within the most recent half of this record. Basal radiocarbon dating (14950—14310 cal yr BP, Supplementary Table S9) suggests that this profile coincides with fire activity from the Antarctic Cold Reversal and into the Holocene. Contemporary studies in Falkland Island human settlement further indicate that estimations of fire activity during this substantial time period are unlikely to originate from historic anthropogenic activity (Europeans arrived in 1764 CE), whilst pre-historic burning disturbance remains uncertain^74,75^. Instead, reconstructions of Holocene fire activity across the Falkland Islands attribute frequent fire disturbance and relative changes in fire intensity to changes in wind speed, fuel type and fuel moisture^21^. In this instance, the notable intensification in fire activity likely coincides with a period of seasonal drying, an influx of shrub vegetation, low precipitation, high winds, and low water table depth^21^. However, the record of fire on the Falklands, and conclusions as to the role of climate and vegetational change, remains spatiotemporally variable^21^.

Our application, here, of Raman palaeo-calorimetry to charcoals preserved in a peat core reflects a preliminary demonstration of the capabilities of this method in unifying reconstructed fire, climate, and vegetational interrelationships. Further research is required to correlate these results with localised palaeoenvironmental change, and more broadly, Holocene fire histories across the Falkland Islands. Consideration must also be paid to the limitations of charcoal taphonomy, discussed in detail elsewhere^76^ and further reinforced by observations, here, of heat flux-dependent charcoal consumption.

More specifically, questions may be raised as to the relevance of limited sampling regimes (e.g., a single core), in tandem with palaeo-calorimetry, when characterising fire activity as an heterogenous macro-disturbance – both spatially and temporally. The autochthonous nature of peatland record accumulation, and inherent heterogeneity in microtopography and ecohydrology, suggest a significant susceptibility to issues of taphonomic bias. Differences in peatland vegetational structures have been accounted for here through the considered application of intact mesocosms. However, spatiotemporal heterogeneity in charcoal records may only be accounted for by expanding sampling and sub-sampling regimes.

The applicability of this palaeo-calorimeter may not be limited solely to applications in palaeofire. Considering microstructural change in carbonaceous materials as a function of energy intensity (energy per unit time) has the potential to solve issues of calibration between different forms of geological maturation^47^. For instance, the formation and maturation of charcoal occurs over minutes, at most (Fig. 1), compared to the multi-millennial maturation experienced by coal macerals^23^. Yet, Raman spectra and parameters are often comparable between both materials^47^. Further research is required to determine the applicability of this to materials under variable geological contexts. Nevertheless, this study offers an exciting prospect for the calibration of thermal maturation in carbon across numerous and varied geological contexts. With respect to the progressive integration of modern and palaeowildfire sciences, however, focus must now turn to the coherence of charcoal microstructure, surface temperatures, and heating conditions. Innovative experimentation, considerate of field-scaling and interdisciplinary aims, is necessary to tackle this complex co-occurrence of thermal exposure, material response, and radiative and convective heating processes.

## Methodology

### Mesocosm sampling

Peatland mesocosm samples were collected from Craigmaud Moss, Aberdeenshire, Scotland (57.61756° N, 2.19281 W [WGS 84]) on the 23rd – 24th June 2022, chosen as a proximal intact northern peatland with a representative vegetational composition. Taxa present at Craigmaud Moss include mosses (*Sphagnum capillifolium*, *Sphagnum* section *Cuspidata*, *Polytrichum* spp., Brown Mosses undiff.), graminoids (*Eriophorum vaginatum*,* Poaceae* spp.), and dwarf shrubs (*Calluna vulgaris*,* Empetrum nigrum*,* Erica tetralix*), with minor additional taxa present (*Cladonia* spp.*).*

Each mesocosm was sampled at random locations across the Craigmaud Moss peatland, according to its composition in relation to five predetermined categories: > 75% mosses (MS_> 75_), > 75% graminoids (GR_> 75_), > 75% dwarf shrubs (DS_> 75_), 25–50% moss-graminoid mix (MSGR_Mix_), and 25–50% dwarf shrub-moss mix (DSMS_Mix_). A further 25–50% dwarf shrub-graminoid mix (DSGR_Mix_) mesocosm was collected from Tor Hill Moss, Perth & Kinross, Scotland (57.24122° N, 3.66804° W [WGS 84]) on the 19th May 2022, given limited presentation of this mesocosm at Craigmaud Moss. These categories were chosen to sufficiently represent the compositional variability of vegetation common to the northern peatlands. For each mesocosm category, four individual samples were collected independently, corresponding to the four different heat flux treatments applied during calorimetry (see below). Detailed taxonomic compositions for each mesocosm sample have been provided in Supplementary Table S1*.* All samples were stored at 4 °C prior to calorimetry.

### Palaeocharcoal sampling

Subfossil and subrecent charcoal assemblages were extracted from thirteen individual depths along a 3.45 m composite peat profile collected from the eastern Falkland Islands (February 2022), using a Russian peat corer with an 8 cm chamber. The core sections were sliced (frozen) into samples of c. 9 cm^3^, each covering c. 1.1 cm of the profile, which were then rinsed with ultrapure water prior to trimming the edges to ensure the removal of potential contamination. Four cubic centimetres of peat per sample were warmed for one hour in 8% NaOH at ~ 50 °C to dissolve humic substances, then sieved through a 180 μm mesh under a gentle stream of deionised water. Following this, the strained macro-remains were transferred into a tray with deionised water. Large, uncharred particles (roots, stems, leaves, etc.) were removed using forceps. Small and light, uncharred particles (fungal, root fragments, etc.) were removed using a 3 ml disposable pipette in repeated steps, until only charcoal (*n* > 25) remained. The application of clean water between repeated steps ensured continued agitation of less dense particles in the water column, permitting rapid absorption. To avoid unintentional biases during manual picking, a disposable macro-pipette (3 ml) was used to randomly isolate macro-charcoal particles from the remaining residues. These charcoal particles were subsequently dried at c. 50 °C, prior to Raman spectroscopy.

### Calorimetry

To eliminate the influence of fuel moisture on combustion behaviour, samples were dried in an oven at 60 °C, for at least 72 h, prior to each experimental run. Using a precision balance (± 0.01 g), the (dry) mass of each sample was recorded prior to- and after calorimetric testing. The cone calorimeter gas analysers and heater were calibrated before experimentation, achieving the required incident heat flux *via* the use of a Schmidt-Boelter type heat flux meter. Samples were positioned at a vertical distance of 25 mm between the top surface of the sample and the underside of the cone heater. During heat flux treatment, samples were held in a 125 mm diameter (30 mm height) porous sample holder to permit airflow, as experienced by natural fuel structures during wildfire.

The heat release rate (HRR) and dynamic smoke production rate were recorded as samples underwent controlled levels of irradiance, employing an external igniter, as described in ISO 5660-1 *as per*^77^ and ASTM E1354 *as per*^78^. Four different incident heat fluxes were applied (20, 40, 60 and 80 kW/m^2^) in order to replicate, systematically, a range of heat fluxes typically experienced during wildfire^64,79^. As soon as flaming ignition occurred, the sample was permitted to burn until flameout, as defined by the termination of a visible flame. Subsequent to flameout, the sample remained undisturbed for 30 s under the respective incident heat flux, before being removed from the calorimeter. For those samples that failed to ignite (flaming), exposure to the conical heater was maintained for up to 10 min prior to removal.

All cone calorimetry was conducted at the University of Edinburgh Fire Research Centre.

### Raman spectroscopy

Following calorimetric experimentation, and the generation of charcoalified materials, all samples were stored at room temperature. Charcoal samples underwent no alterative chemical or mechanical treatments and were selected and isolated for Raman spectroscopic analysis manually using clean forceps. Over 25 charcoal pieces were selected randomly from each charred mesocosm, as a representative sample for Raman spectroscopic analysis, using a random number generator and coordinate selection grid.

During Raman spectral acquisition, a random selection of charcoal pieces from each mesocosm subsample was transferred onto a glass microscopy slide. From these charcoals, one spectrum was collected per charcoal piece, across 25 individual pieces per mesocosm selected at random. This sampling protocol was applied to ensure that the bulk variability of charcoal material within each sample was accounted for, and that no biases or limitations were placed on the types and/or condition of material chosen for spectral acquisition. Sites for spectral acquisition were selected from charcoal surfaces with clear, highly reflective cellular features. This is typically indicative of a flat material surface that is conducive to a strong Raman response.

Details regarding the spectroscopic setup utilised throughout this study have been summarised in Supplementary Table S2. The Raman methodology in application here remains consistent with efficient, effective, and reliable protocols recorded within existing literature^80^, including those specific to charcoalified plant material (e.g.,^21,43,46^).

To ensure the efficient, accessible, and reproducible generation of quantitative spectral data, a standardised method of automated deconvolution was applied to this dataset. Designed for and performed within MATLAB (v. R2021a), the automated deconvolution code applied here utilises a ‘peak fit’ function base code^81,82^, incorporated into a secondary deconvolution code, as developed and applied by Schito et al.^83,84^. The secondary code has been modified further here (with permission) to best replicate established practices in (manual) spectral deconvolution of charcoal spectra^85,86^. This includes the application of a smoothing function, a linear baseline anchored between 1050 and 1150 cm^−1^ and 1650–1750 cm^−1^, and a pseudo-Voigt fit (50% Gaussian-Lorentzian) to bands ‘D’ (~ 1350 cm^−1^) and ‘G’ (~ 1585 cm^−1^).

Automated deconvolution provides the following spectral parameters: band position (cm^−1^), band width (‘*FWHM*’, cm^−1^), band intensity ratio (‘*R1*’), band width ratio (‘*FWHMRa*’), band area ratio (‘*A*_*D*_*/A*_*G*_’), and Raman band separation (‘*RBS*’, cm^−1^).

Raman analyses, as applied to palaeocharcoal material from the eastern Falkland Islands peat profile, remained consistent with protocols outlined above. In accordance with this, fragments of charcoal isolated from the relevant peat profile were placed onto a glass microscopy slide, and 25 spectral measurements (per sample) were collected at random across individual fragments within the bulk mass of particles.

### Statistical analyses

One-way ANOVA and Bayesian ANOVA tests were performed in JASP (v. 0.17.1). PCA ordination, as well as permutational multivariate analyses of variance (PERMANOVA) were performed in R 4.2.3 (*as per*^87^) using the *vegan*^88^, *readx1*, *factoextra*, *ggfortify*, and *ggrepel* packages. PERMANOVA, as applied here, incorporates the ‘Adonis’ function and Bray-Curtis distance matrix over 10,000 iterations - further details of which are available within the derivative ‘R’ code, provided in *Supplementary Data*. Linear regression modelling was also undertaken in R 4.2.3.

## Supplementary Information

Supplementary Material 1.

## Data Availability

The datasets generated during and/or analysed during the current study are available from the corresponding author on reasonable request.
