# Supplementary material for: A novel proxy for energy flux in multi-era wildfire reconstruction
Source: Sci Rep. 2024 Nov 2;14:26409. doi: 10.1038/s41598-024-78219-3 (PMC11531590; doi:10.1038/s41598-024-78219-3)
Supplement: Supplementary file 1 — Supplementary Material 1. [file 41598_2024_78219_MOESM1_ESM.docx]

*Supplementary Data* pertaining to

**A novel proxy for energy flux in multi-era wildfire reconstruction**

Thomas Theurer^1*^, Dmitri Mauquoy^1^, Rory Hadden^2^, David Muirhead^1^, Zakary Campbell-Lochrie^2^, Sergio Vargas Córdoba^2^, Clemens von Scheffer^1^, and Daniel Coathup^1^.

^1^ School of Geosciences, University of Aberdeen, Aberdeen, United Kingdom.

^2^ School of Engineering, University of Edinburgh, Edinburgh, United Kingdom.

^*^ Corresponding Author: Thomas Theurer (thomas.theurer2@abdn.ac.uk)

1. **Mesocosm Sample Composition**

**Supplementary Table S1;** A table detailing the taxa present within each mesocosm sample, a qualitative measure of their proportion (Major: MAJ, Minor: MIN), and further information regarding the field moisture content upon collection (FMC) and source locality.

| Mesocosm | Sample Number [Heat Flux Treatment] | Locality | FMC (%) | Taxa Present | | | | | | | | | |
| --- | --- | --- | --- | --- | --- | --- | --- | --- | --- | --- | --- | --- | --- |
|  |  |  |  | Mosses | | | | Graminoids | | Dwarf Shrubs | | | Lichens |
|  |  |  |  | *Sphagnum capillifolium* | *Sphagnum section Cuspidata* | Brown Moss  (undiff.) | *Polytrichum* spp*.* | *Eriophorum vaginatum* | *Poaceae* spp. | *Calluna vulgaris* | *Empetrum nigrum* | *Erica tetralix* | *Cladonia* spp. |
| ≥ 75 % Mosses | A1 [20] | Craigmaud Moss | 82.8 |  | MAJ |  |  |  |  |  |  |  |  |
|  | A3 [40] |  | 15.2 | MAJ |  |  |  | MIN |  |  |  | MIN |  |
|  | A5 [60] |  | 30.8 | MAJ |  |  |  | MIN |  |  |  | MIN |  |
|  | A7 [80] |  | 39.0 |  |  | MAJ |  | MIN |  | MIN |  |  |  |
| ≥ 75 % Graminoids | B1 [20] |  | 6.4 |  |  | MIN |  |  | MAJ | MIN |  |  |  |
|  | B4 [40] |  | 55.6 |  |  | MIN |  |  | MAJ |  |  |  |  |
|  | B7 [60] |  | 24.4 |  |  |  |  | MAJ |  |  |  | MIN |  |
|  | B8 [80] |  | 20.2 |  |  | MIN |  | MIN | MAJ |  |  |  |  |
| ≥ 75 % Dwarf Shrubs | C3 [20] |  | 4.1 |  |  | MIN |  | MIN |  | MAJ |  |  |  |
|  | C4 [40] |  | 8.2 |  |  | MIN |  | MIN |  | MIN | MAJ |  |  |
|  | C6 [60] |  | 2.1 |  |  | MIN |  | MIN |  | MAJ |  |  |  |
|  | C7 [80] |  | 2.8 |  |  | MIN |  | MIN |  | MAJ |  | MIN | MIN |
| 25 − 50 % Mosses & Graminoids | D1 [20] |  | 5.8 |  |  | MAJ |  | MAJ |  | MIN |  |  |  |
|  | D3 [40] |  | 1.2 |  |  | MAJ |  | MAJ | MIN |  |  |  | MIN |
|  | D4 [60] |  | 1.2 |  |  | MAJ |  | MAJ |  | MIN |  |  |  |
|  | D6 [80] |  | 2.6 |  |  | MAJ |  | MAJ |  | MIN | MIN |  |  |
| 25 − 50 % Dwarf Shrubs & Graminoids | E1 [20] |  | 14.8 |  |  | MIN |  | MAJ |  |  | MAJ |  |  |
|  | E2 [40] |  | 3.8 |  |  | MIN |  | MAJ |  |  | MAJ |  |  |
|  | E4 [60] |  | 15.4 |  |  | MIN |  | MAJ |  |  | MAJ |  |  |
|  | E7 [80] |  | 3.3 |  |  |  |  | MAJ | MIN |  |  | MAJ |  |
| 25 − 50 % Dwarf Shrubs & Mosses | F1 [20] | Tor Hill Moss | 29.9 | MAJ |  |  |  | MIN |  | MAJ |  |  |  |
|  | F3 [40] |  | 34.6 | MAJ |  | MIN |  | MIN |  | MAJ |  |  |  |
|  | F4 [60] |  | 33.9 |  |  | MAJ |  |  |  | MAJ |  |  | MIN |
|  | F7 [80] |  | 31.8 |  |  | MAJ |  |  |  | MAJ |  |  | MIN |

1. **Raman Spectroscopic Setup**

**Supplementary Table S2;** A table detailing the hardware specifications and spectral acquisition setup parameters applied during this study.

| Component | Sub-component | Details |
| --- | --- | --- |
| Spectrometer | Model | *Renishaw* ‘InVia Reflex’ Raman spectrometer |
| Microscope | Model | *Leica* DM2700M |
|  | Objective Magnification | ×50 |
| Laser | Source | Green diode |
|  | Wavelength | 514.5 nm |
|  | Applied Power | <0.3 mW (1% Total output) |
|  | Spot Size | 1 – 2 µm |
| Calibration | Reference Peak | Silicon (520.5 cm^-1^) |
|  | Reference Offset | As required |
|  | Beamsteer | As required |
| Spectral Acquisition | Grating | 2400 l/mm (Visible spectrum) |
|  | Scan Centre | 1400 cm^-1^ |
|  | Scan Range | ~1100 – 1700 cm^-1^ |
|  | Scan Resolution | ~3 cm^-1^ |
|  | Additional Functions | Cosmic ray removal |
|  | N^o.^ Acquisitions | 3 |
|  | Duration of Exposure | 5s per acquisition (15s Total exposure) |

1. **Permutational Multivariate Analysis of Variance (PERMANOVA) ‘R’ Code**

*#PCA in vegan*library(vegan)
library(readxl)
library(factoextra)
library(ggfortify)
library(ggrepel)

*#Read in data*ConeC_Energy_RBS
ConeC_Energy_species

*#preparation*
cor(ConeC_Energy_RBS)
mean(cor(ConeC_Energy_RBS))

pca <- rda(decostand(ConeC_Energy_RBS, method = "hellinger"), scale = TRUE, CENTER = TRUE)
pca
summary(pca)
scores(pca)

*#Eigenvalues*

pca.data.PCs <- as.data.frame(pca$CA$eig)
pca.data.PC1and2 <- as.data.frame(pca$CA$eig[1:2])
pca.data.species <- as.data.frame(pca$CA$v)
pca.data.sites <- as.data.frame(pca$CA$u)

biplot(pca, scaling = "symmetric")

*#Method 2_prcomp package*
pca.prcomp <- prcomp(ConeC_Energy_RBS, scale = TRUE, center = TRUE)
pca.prcomp

pca.prcomp.h <-decostand(ConeC_Energy_RBS, "hellinger")
pca.prcomp <- prcomp(pca.prcomp.h, scale = TRUE, center = TRUE)
pca.prcomp

summary(pca.prcomp)

*#Extract eigenvalues*
eig.val <- get_eigenvalue(pca.prcomp)
eig.val

str(pca.prcomp)
pca.prcomp$x
varespec.PCAs <- cbind(ConeC_Energy_RBS, pca.prcomp$x[,1:2])

*#package factoextra*
fviz_eig(pca.prcomp)

biplot(pca.prcomp, scaling = "symmetric")

fviz_pca_biplot(pca.prcomp, repel = TRUE,
 col.var = "#2E9FDF", col.ind = "#696969")

pca.prcomp.data <- data.frame(pca.prcomp$x)
pca.prcomp.data$plotx <- pca.prcomp.data[,1]
pca.prcomp.data$ploty <- pca.prcomp.data[,2]
species = as.character(ConeC_Energy_species$Species)
pca.prcomp.data$group <- species

*#PCA with Mesocosm overlay*pca.prcomp.plot <- ggplot(pca.prcomp.data, aes(x=plotx, y=ploty, color=group)) +
 geom_point() +
 ggtitle("PCA with mesocosm") +
 xlab("PC1") + ylab("PC2") +
 coord_fixed(ratio = 1) +
 theme_bw() + theme(aspect.ratio=1) + theme(panel.grid = element_blank()) +
 stat_ellipse(geom = "polygon", aes(fill = after_scale(alpha(colour, 0.3))),
 data= pca.prcomp.data[pca.prcomp.data$group != "versicolor",])
pca.prcomp.plot

distance <- vegdist(ConeC_Energy_RBS, method = "bray")
set.seed(19672605)
adonis2(distance ~ Species, data = ConeC_Energy_species, permutations=10000)

1. **Descriptive Statistics**

**Supplementary Table S3;** A table providing median, mean, minimum, maximum, and standard deviation values for those parameter-mesocosm combinations considered most reliable (R1, RBS, and A_D_/A_G_ for DS_>75_ and MSGR_Mix_ mesocosms) in characterising an imposed heat flux during experimental calorimetry.

| Mesocosm | Raman Parameter | Heat Flux Applied (kWm^2^) | *n* | Descriptive Statistics | | | | |
| --- | --- | --- | --- | --- | --- | --- | --- | --- |
|  |  |  |  | Median | Mean | Minimum | Maximum | S.D. |
| > 75% Dwarf Shrub | R1 | 20 | 25 | 0.590 | 0.583 | 0.480 | 0.680 | 0.065 |
|  |  | 40 | 25 | 0.660 | 0.685 | 0.540 | 0.980 | 0.103 |
|  |  | 60 | 25 | 0.730 | 0.729 | 0.560 | 0.930 | 0.087 |
|  |  | 80 | 25 | 0.730 | 0.739 | 0.650 | 0.890 | 0.061 |
|  | RBS | 20 | 25 | 216.640 | 215.968 | 195.690 | 226.200 | 7.451 |
|  |  | 40 | 25 | 222.030 | 222.633 | 215.490 | 232.280 | 4.160 |
|  |  | 60 | 25 | 232.370 | 231.172 | 217.870 | 240.460 | 5.240 |
|  |  | 80 | 25 | 232.300 | 231.871 | 220.660 | 237.570 | 4.136 |
|  | A_D_/A_G_ | 20 | 25 | 1.600 | 1.577 | 1.060 | 1.860 | 0.194 |
|  |  | 40 | 25 | 1.830 | 1.826 | 1.550 | 2.080 | 0.138 |
|  |  | 60 | 25 | 1.990 | 2.000 | 1.760 | 2.250 | 0.125 |
|  |  | 80 | 25 | 2.050 | 2.051 | 1.880 | 2.220 | 0.077 |
| 25 – 50% Moss-Graminoid Mix | R1 | 20 | 25 | 0.610 | 0.627 | 0.520 | 0.920 | 0.091 |
|  |  | 40 | 25 | 0.690 | 0.704 | 0.580 | 0.840 | 0.078 |
|  |  | 60 | 25 | 0.750 | 0.769 | 0.570 | 0.980 | 0.118 |
|  |  | 80 | 25 | 0.760 | 0.764 | 0.630 | 0.850 | 0.053 |
|  | RBS | 20 | 25 | 220.280 | 219.828 | 211.660 | 231.000 | 3.812 |
|  |  | 40 | 25 | 227.950 | 226.936 | 216.260 | 231.010 | 2.996 |
|  |  | 60 | 25 | 224.260 | 224.387 | 214.690 | 234.440 | 4.567 |
|  |  | 80 | 25 | 231.080 | 230.566 | 222.610 | 237.480 | 3.599 |
|  | A_D_/A_G_ | 20 | 25 | 1.740 | 1.726 | 1.390 | 2.270 | 0.174 |
|  |  | 40 | 25 | 1.820 | 1.831 | 1.650 | 2.060 | 0.105 |
|  |  | 60 | 25 | 1.970 | 1.942 | 1.490 | 2.190 | 0.168 |
|  |  | 80 | 25 | 2.080 | 2.081 | 1.910 | 2.190 | 0.077 |

1.
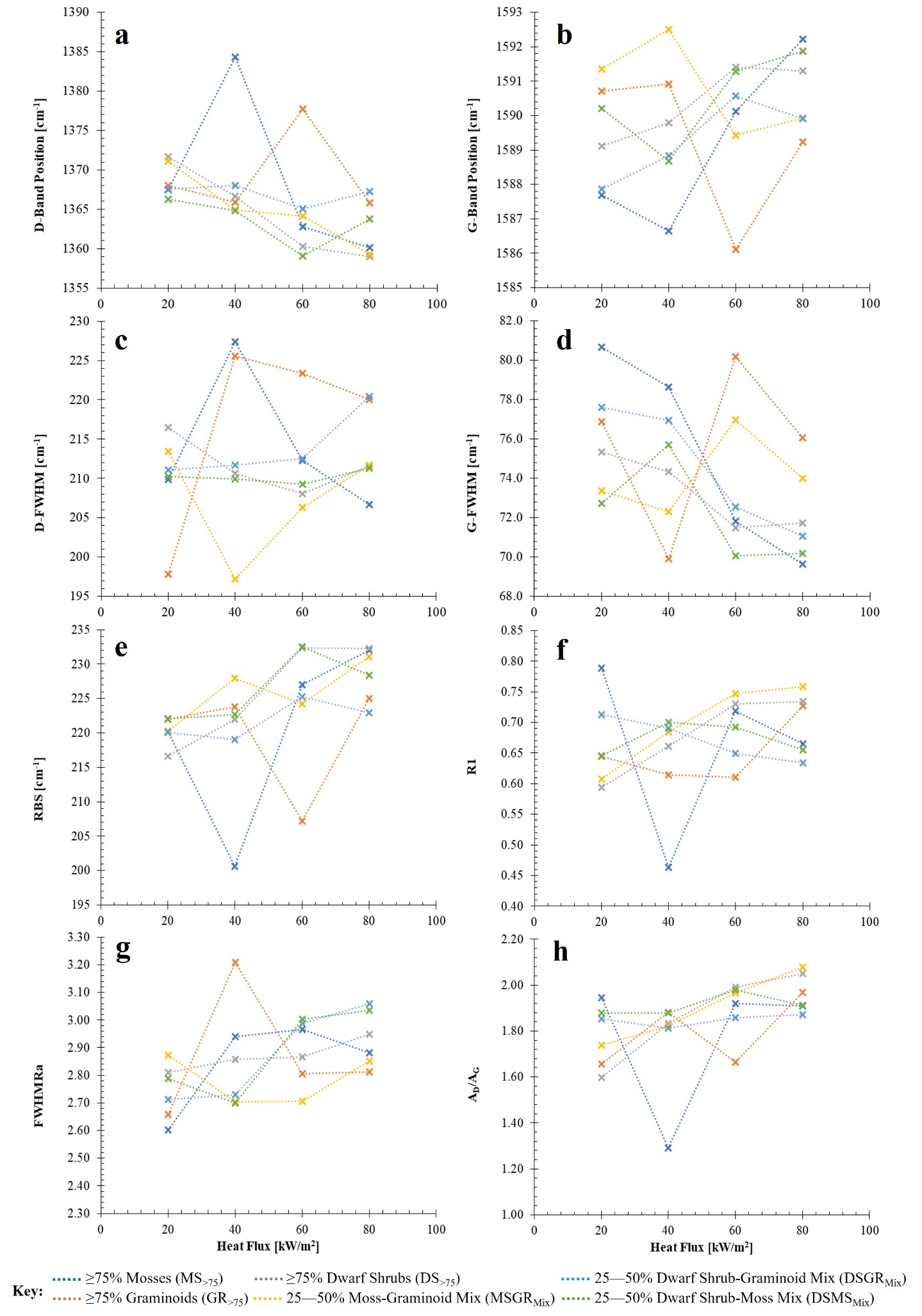
**Raman Parametric Trends with Heat Flux**

**Supplementary Figure S1;** Data plots indicating the behaviour of Raman parameters measured from charcoals, as generated under increasing heat flux during cone calorimetry from six varying peatland mesocosm (i.e., surface plant mix) compositions. Note, key and x-axes refer to all graphs presented (a-h). Those Raman parameters measured include: (a) D-band position; (b) G-band position; (c) D-FWHM; (d) G-FWHM; (e) RBS; (f) R1; (g) FWHMRa; and (h) A_D_/A_G_. For additional details as to the nature of these parameters, the reader is referred to the original article.

1. **ANOVA, Levene’s, & Post Hoc Pairwise Comparison**

**Supplementary Table S4;** A table displaying the results of ANOVA, Equality of Variance, and Tukey/Games-Howell Post Hoc testing (dependent upon the results for variance equality) for those parameter-mesocosm combinations considered most reliable (R1, RBS, and A_D_/A_G_ for DS_>75_ and MSGR_Mix_ mesocosms) in characterising an imposed heat flux during experimental calorimetry. Here, ‘NS’ refers to a statistical result that is not significant, ‘*df*’ corresponds to degrees of freedom, and ‘ɳ^2^’ represents the eta-squared effect size.

| Mesocosm | Parameter | *df1, df2* | ANOVA | | | Equality of Variance (Levene’s Test) | | Post Hoc (Tukey/Games-Howell) | |
| --- | --- | --- | --- | --- | --- | --- | --- | --- | --- |
|  |  |  | *F* | *p* | ɳ^2^ | *F* | *p* | Heat Flux Pairwise Comparison | *p* |
| > 75% Dwarf Shrub | R1 | 3, 96 | 19.358 | <0.001 | 0.377 | 1.813 | NS | 20 – 40 | <0.001 |
|  |  |  |  |  |  |  |  | 20 – 60 | <0.001 |
|  |  |  |  |  |  |  |  | 20 – 80 | <0.001 |
|  |  |  |  |  |  |  |  | 40 – 60 | NS |
|  |  |  |  |  |  |  |  | 40 – 80 | NS |
|  |  |  |  |  |  |  |  | 60 – 80 | NS |
|  | RBS |  | 48.791 | <0.001 | 0.604 | 3.823 | <0.05 | 20 – 40 | <0.01 |
|  |  |  |  |  |  |  |  | 20 – 60 | <0.001 |
|  |  |  |  |  |  |  |  | 20 – 80 | <0.001 |
|  |  |  |  |  |  |  |  | 40 – 60 | <0.001 |
|  |  |  |  |  |  |  |  | 40 – 80 | <0.001 |
|  |  |  |  |  |  |  |  | 60 – 80 | NS |
|  | A_D_/A_G_ |  | 58.581 | <0.001 | 0.647 | 4.819 | <0.01 | 20 – 40 | <0.001 |
|  |  |  |  |  |  |  |  | 20 – 60 | <0.001 |
|  |  |  |  |  |  |  |  | 20 – 80 | <0.001 |
|  |  |  |  |  |  |  |  | 40 – 60 | <0.001 |
|  |  |  |  |  |  |  |  | 40 – 80 | <0.001 |
|  |  |  |  |  |  |  |  | 60 – 80 | NS |
| 25 – 50% Moss-Graminoid Mix | R1 |  | 14.113 | <0.001 | 0.306 | 3.779 | <0.05 | 20 – 40 | <0.05 |
|  |  |  |  |  |  |  |  | 20 – 60 | <0.001 |
|  |  |  |  |  |  |  |  | 20 – 80 | <0.001 |
|  |  |  |  |  |  |  |  | 40 – 60 | NS |
|  |  |  |  |  |  |  |  | 40 – 80 | <0.05 |
|  |  |  |  |  |  |  |  | 60 – 80 | NS |
|  | RBS |  | 35.536 | <0.001 | 0.526 | 1.195 | NS | 20 – 40 | <0.001 |
|  |  |  |  |  |  |  |  | 20 – 60 | <0.001 |
|  |  |  |  |  |  |  |  | 20 – 80 | <0.001 |
|  |  |  |  |  |  |  |  | 40 – 60 | NS |
|  |  |  |  |  |  |  |  | 40 – 80 | <0.01 |
|  |  |  |  |  |  |  |  | 60 – 80 | <0.001 |
|  | A_D_/A_G_ |  | 30.738 | <0.001 | 0.490 | 2.529 | NS | 20 – 40 | <0.05 |
|  |  |  |  |  |  |  |  | 20 – 60 | <0.001 |
|  |  |  |  |  |  |  |  | 20 – 80 | <0.001 |
|  |  |  |  |  |  |  |  | 40 – 60 | <0.05 |
|  |  |  |  |  |  |  |  | 40 – 80 | <0.001 |
|  |  |  |  |  |  |  |  | 60 – 80 | <0.01 |

1. **Bayesian ANOVA**

**Supplementary Table S5;** A table displaying the results of Bayesian ANOVA modelling for those parameter-mesocosm combinations considered most reliable (R1, RBS, and A_D_/A_G_ for DS_>75_ and MSGR_Mix_ mesocosms) in characterising an imposed heat flux during experimental calorimetry. Here, P(M), P(M|Data), BF_M_, and BF_10_ refer to the prior model probability, posterior model probability, posterior model odds, and comparative bayes factor, respectively. For MSGR_Mix_ Bayesian models, posterior model odds and bayes factors are presented as their logarithmic function derivative, i.e., log(BF_M_) and log(BF_10_), respectively.

| Mesocosm | Model | P(M) | P(M\|Data) | BF_M_ | **BF_10_** | Error (%) |
| --- | --- | --- | --- | --- | --- | --- |
| > 75% Dwarf Shrub | Null | 0.500 | 6.509×10^-8^ | 6.509×10^-8^ | 1.000 | - |
|  | R1 | 0.500 | 1.000 | 1.536×10^7^ | **1.536×10^7^** | 0.001 |
| > 75% Dwarf Shrub | Null | 0.500 | 6.940×10^-17^ | 6.940×10^-17^ | 1.000 | - |
|  | RBS | 0.500 | 1.000 | 1.441×10^16^ | **1.441×10^16^** | 0.001 |
| > 75% Dwarf Shrub | Null | 0.500 | 3.448×10^-19^ | 3.448×10^-19^ | 1.000 | - |
|  | A_D_/A_G_ | 0.500 | 1.000 | 2.900×10^18^ | **2.900×10^18^** | 0.002 |
| 25 – 50% Moss-Graminoid Mix | Null | 0.500 | 7.579×10^-6^ | -11.790 | 0.000 | - |
|  | R1 | 0.500 | 1.000 | 11.790 | **11.790** | 6.174×10^-4^ |
| 25 – 50% Moss-Graminoid Mix | Null | 0.500 | 2.671×10^-13^ | -28.951 | 0.000 | - |
|  | RBS | 0.500 | 1.000 | 28.951 | **28.951** | 0.001 |
| 25 – 50% Moss-Graminoid Mix | Null | 0.500 | 7.763×10^-12^ | -25.582 | 0.000 |  |
|  | A_D_/A_G_ | 0.500 | 1.000 | 25.582 | **25.582** | 1.009×10^-5^ |

1. **Bayesian Model Averaged Posterior Testing**

**Supplementary Table S6;** A table displaying the results of model averaged posterior testing of Bayesian models as presented in Supplementary Table S5. Credible intervals at each level (i.e., heat flux) for the respective parameter-mesocosm combinations are shown, with those in **bold** denoting credible interval overlap.

| Mesocosm | Model | Variable | Level | Mean | Standard Deviation | 95% Credible Intervals | |
| --- | --- | --- | --- | --- | --- | --- | --- |
|  |  |  |  |  |  | Lower | Upper |
| > 75% Dwarf Shrub | R1 | Intercept | | 0.684 | 0.008 | 0.667 | 0.699 |
|  |  | Heat Flux | 20 | -0.094 | 0.014 | -0.123 | -0.066 |
|  |  |  | 40 | 0.001 | 0.014 | -0.027 | **0.029** |
|  |  |  | 60 | 0.042 | 0.014 | **0.014** | **0.069** |
|  |  |  | 80 | 0.052 | 0.014 | **0.023** | 0.080 |
| > 75% Dwarf Shrub | RBS | Intercept | | 225.406 | 0.549 | 224.123 | 226.364 |
|  |  | Heat Flux | 20 | -9.178 | 0.950 | -11.103 | -7.296 |
|  |  |  | 40 | -2.691 | 0.932 | -4.540 | -0.810 |
|  |  |  | 60 | 5.594 | 0.940 | 3.714 | **7.486** |
|  |  |  | 80 | 6.275 | 0.937 | **4.358** | 8.109 |
| > 75% Dwarf Shrub | A_D_/A_G_ | Intercept | | 1.863 | 0.014 | 1.831 | 1.891 |
|  |  | Heat Flux | 20 | -0.280 | 0.024 | -0.328 | -0.232 |
|  |  |  | 40 | -0.037 | 0.024 | -0.086 | 0.010 |
|  |  |  | 60 | 0.134 | 0.024 | 0.084 | **0.182** |
|  |  |  | 80 | 0.183 | 0.025 | **0.132** | 0.232 |
| 25 – 50% Moss-Graminoid Mix | R1 | Intercept | | 0.716 | 0.009 | 0.697 | 0.732 |
|  |  | Heat Flux | 20 | -0.081 | 0.016 | -0.113 | -0.049 |
|  |  |  | 40 | -0.011 | 0.015 | **-0.042** | **0.018** |
|  |  |  | 60 | 0.049 | 0.015 | **0.018** | **0.079** |
|  |  |  | 80 | 0.044 | 0.015 | **0.013** | **0.074** |
| 25 – 50% Moss-Graminoid Mix | RBS | Intercept | | 225.430 | 0.384 | 224.162 | 225.941 |
|  |  | Heat Flux | 20 | -5.389 | 0.667 | -6.722 | -4.059 |
|  |  |  | 40 | 1.442 | 0.656 | **0.117** | **2.737** |
|  |  |  | 60 | -1.001 | 0.653 | **-2.322** | **0.281** |
|  |  |  | 80 | 4.947 | 0.663 | 3.585 | 6.246 |
| 25 – 50% Moss-Graminoid Mix | A_D_/A_G_ | Intercept | | 1.895 | 0.014 | 1.863 | 1.919 |
|  |  | Heat Flux | 20 | -0.162 | 0.024 | -0.211 | -0.115 |
|  |  |  | 40 | -0.061 | 0.024 | -0.108 | -0.013 |
|  |  |  | 60 | 0.045 | 0.024 | -0.004 | 0.091 |
|  |  |  | 80 | 0.179 | 0.025 | 0.129 | 0.228 |

1. **Spearman’s Rank Correlation & Linear Regression**

**Supplementary Table S7;** A table displaying the results of Spearman’s rank correlations between whole data median Raman values (R1**_∑_**, RBS**_∑_**, A_D_/A_G_**_∑_**) and data collected during calorimetry, relating to combustive behaviour. Significant correlations are highlighted in **bold**.

| Correlation | | | Spearman’s rho | *p* |
| --- | --- | --- | --- | --- |
| **Mass Loss [%]** | *vs.* | **Total Incident Energy** | **0.698** | **<0.001** |
| Mass Loss [%] |  | Energy Release | 0.150 | 0.483 |
| Mass Loss [%] |  | Total Carbon Yield | 0.385 | 0.064 |
| **Mass Loss [%]** |  | **RBS_∑_** | **0.584** | **0.003** |
| Mass Loss [%] |  | R1**_∑_** | 0.285 | 0.178 |
| **Mass Loss [%]** |  | **A_D_/A_G∑_** | **0.568** | **0.004** |
| **Total Incident Energy** |  | **Energy Release** | **0.416** | **0.044** |
| **Total Incident Energy** |  | **Total Carbon Yield** | **0.603** | **0.002** |
| **Total Incident Energy** |  | **RBS_∑_** | **0.745** | **<0.001** |
| Total Incident Energy |  | R1**_∑_** | 0.299 | 0.155 |
| **Total Incident Energy** |  | **A_D_/A_G∑_** | **0.512** | **0.011** |
| Energy Release |  | Total Carbon Yield | -0.011 | 0.959 |
| Energy Release |  | RBS**_∑_** | 0.323 | 0.124 |
| Energy Release |  | R1**_∑_** | 0.004 | 0.985 |
| Energy Release |  | A_D_/A_G_**_∑_** | 0.025 | 0.908 |
| Total Carbon Yield |  | RBS**_∑_** | 0.313 | 0.136 |
| Total Carbon Yield |  | R1**_∑_** | 0.014 | 0.950 |
| Total Carbon Yield |  | A_D_/A_G_**_∑_** | 0.224 | 0.291 |
| **RBS_∑_** |  | **R1_∑_** | **0.504** | **0.013** |
| **RBS_∑_** |  | **A_D_/A_G∑_** | **0.787** | **<0.001** |
| **R1_∑_** |  | **A_D_/A_G∑_** | **0.792** | **<0.001** |

1. **Generalised Least-squares Regression**
2. **RBS_∑_** explained 29.1% of the variance in the **Energy Released** data.

(Adjusted R_2_ = 0.291, F(1, 22) = 10.43, p<0.01). b = 3.448 (SE = 1.068), p<0.01.

Intercept = -621.762

1. **RBS_∑_** explained 16.6% of the variance in the **Total Incident Energy** data.

(Adjusted R_2_ = 0.166, F(1, 22) = 5.571, p<0.05). b = 0.514 (SE = 0.218), p<0.05.

Intercept = -104.04

1. **Holocene Fire Calorimetry (Falkland Islands Peatland)**

**Supplementary Table S8;** A table presenting the results of universal palaeo-calorimetry, applied to palaeocharcoal material sampled from Holocene peat deposits from the Falkland Islands. Estimations of palaeofire energy release (kJ) and total incident energy (MJ) have been generated through the application of palaeo-calorimetry equations (1) and (2) (see main article), respectively. The colour scheme, applied here to median energy release (kJ) and total incident energy (MJ) values, corresponds to a relative indication of intensity, denoted by lowest (dark green), moderate (yellow), and highest values (dark orange).

| Sample | Depth (m) | Energy Released (kJ) | | | | Total Incident Energy (MJ) | | | |
| --- | --- | --- | --- | --- | --- | --- | --- | --- | --- |
|  |  | Median | Lower (95%) Confidence Interval | Upper (95%) Confidence Interval | Standard Deviation | Median | Lower (95%) Confidence Interval | Upper (95%) Confidence Interval | Standard Deviation |
| GG_RE_0 | 0 (Surface) | 157.9 | 132.8 | 183.0 | 33.50 | 12.09 | 8.87 | 15.30 | 4.99 |
| GG_1A_18 | 0.216 | 176.0 | 142.9 | 209.1 | 14.85 | 14.78 | 10.12 | 19.45 | 2.21 |
| GG_1B_20 | 0.64 | 179.5 | 144.6 | 214.5 | 11.71 | 15.31 | 10.28 | 20.35 | 1.74 |
| GG_1B_32 | 0.79 | 171.2 | 140.4 | 202.0 | 16.15 | 14.07 | 9.87 | 18.28 | 2.41 |
| GG_1A_60 | 1.03 | 163.2 | 136.0 | 190.4 | 25.13 | 12.87 | 9.34 | 16.41 | 3.75 |
| GG_1B_48 | 1.27 | 164.9 | 137.0 | 192.9 | 21.48 | 13.13 | 9.47 | 16.80 | 3.20 |
| GG_1B_64 | 1.47 | 152.4 | 129.1 | 175.8 | 37.43 | 11.27 | 8.26 | 14.28 | 5.58 |
| GG_1A_94 | 1.73 | 131.4 | 110.4 | 152.3 | 22.27 | 8.14 | 4.42 | 11.85 | 3.32 |
| GG_1B_94 | 2.134 | 148.7 | 126.4 | 171.1 | 26.24 | 10.72 | 7.75 | 13.69 | 3.91 |
| GG_1B_142 | 2.63 | 155.0 | 130.8 | 179.1 | 47.75 | 11.65 | 8.56 | 14.74 | 7.12 |
| GG_1A_143 | 3.02 | 158.8 | 133.3 | 184.3 | 36.50 | 12.22 | 8.96 | 15.48 | 5.44 |
| GG_1B_154 | 3.17 | 154.0 | 130.2 | 177.8 | 36.05 | 11.50 | 8.45 | 14.56 | 5.37 |
| GG_1A_205 | 3.45 | 148.2 | 126.0 | 170.4 | 18.89 | 10.64 | 7.68 | 13.61 | 2.82 |

1. **Falkland Island Peatland Core Radiocarbon Dating**

**Supplementary Table S9;** Experimental and derivative detail as to the basal radiocarbon date from the Falkland Islands’ peat core profile. When isolating the relevant material for dating, an initial peat sample was warmed in NaOH, then sieved through a 180µm mesh with distilled water. Charred graminoid leaf bases and stems (in distilled water) were then picked under a microscope, dried, and prepared carbon targets measured using accelerator mass spectrometry at the *Chrono Centre* Radiocarbon Lab, Queen’s University, Belfast, UK. The calibrated age, as detailed here and in the original article, was determined by application of the Southern Hemisphere calibration curve^1^ in ‘R’ programme *rbacon*^2^. Here ‘years BP’ refers to ‘years before present’.

| Lab. Code | Material | ^14^C Age (years BP) | ± (years) | F^14^C | ± | Graphite (mg) | Calibrated Age  (cal years BP) |
| --- | --- | --- | --- | --- | --- | --- | --- |
| UBA-49617 | Charred graminoid leaf bases and stems | 12485 | 50 | 0.2114 | 0.0013 | 0.674 | 14950 – 14310 |

[1] Hogg, A. G. *et al*. SHCal20 Southern Hemisphere Calibration, 0–55,000 Years cal BP. *Radiocarbon.* **62**(4), 759-778; 10.1017/RDC.2020.59 (2020).

[2] Blaauw, M. & Christen J. A. Flexible paleoclimate age-depth models using an autoregressive gamma process. *Bayesian Anal.* 6(3), 457-474; 10.1214/11-BA618 (2011).
